# Magnet-Retained Two-Mini-Implant Overdenture: Clinical and Mechanical Consideration

**DOI:** 10.3390/dj4040035

**Published:** 2016-10-10

**Authors:** Yuichi Ishida, H. S. Kiran Kumar, Takaharu Goto, Megumi Watanabe, Rudi Wigianto, Tetsuo Ichikawa

**Affiliations:** 1 Department of Oral & Maxillofacial Prosthodontics, Tokushima University, Graduate School of Biomedical Sciences, 3-18-15, Kuramoto, Tokushima 770-8504, Japan; junchan@tokushima-u.ac.jp (Y.I.); tak510@tokushima-u.ac.jp (T.G.); megwat@tokushima-u.ac.jp (M.W.); 2 Department of Prosthodontics, Sri Hasanamba Dental College and Hospital, Vidyanagar, Hassan, Karnataka 537201, India; dr.kiranhs@gmail.com; 3 Private clinic, Jl, Hayam Wuruk #96, Denpasar, Bali 80235, Indonesia; rwigianto@yahoo.com; 4 Visiting lecturer at Faculty of Dentistry, Hang Tuah University, Surabaya 60111, Indonesia

**Keywords:** mini-implant, overdenture, magnet, biomechanical

## Abstract

Two-implant overdentures have become the accepted treatment for restoring mandibular edentulism. The dimensions of regular implants sometimes limit their use, such as in the case of narrow ridges. Mini-implants with reduced diameters (less than 3.0 mm) enable insertion into narrow ridges. A magnet-retained two-mini-implant overdenture system was developed and is described in this paper. Additionally, we describe a clinical mandibular procedure using the system and evaluate its biomechanical performance.

## 1. Introduction

After the Mcgill and York consensus, two-implant overdentures (2-IOD) have become the accepted treatment of choice for restoring mandibular edentulism due to the favorable results, including improvement in oral function and patient satisfaction [1,2,3]. However, selection of the appropriate type of implant and connectors to assure retention and stability are still controversial due to the resorption pattern of the mandibular arch.

Despite the advantages of two-implant overdentures, they may show limitations in some cases. Patients who could benefit from implant therapy may reject them due to fear of oral surgery or other psychological issues [4]. Cost plays an important role for some patients, systemic diseases can restrict operative procedures, and a long duration of treatment is undesirable, especially for the elderly [5]. Furthermore, the dimensions of standard implants limit their use, particularly in cases involving narrow ridges [6].

Mini-implants are an alternative to standard fixtures; their reduced diameter (less than 3.0 mm) enables insertion into narrow ridges [7,8,9]. The main advantages of mini-implants are that they are minimally invasive, cost-effective, and require a short treatment duration; this motivates patients to choose implants for the stabilization of their dentures [10]. The use of proper attachments can enhance retention and stability of the overdenture.

Several types of attachments are employed for implant overdenture, such as splinting (bar-clip constructions with various bar-shape designs, compared with various ball-type attachments, magnet attachments, and attachments with telescopic copings) [11,12]. Splinting by bar-clip is most common, but ball and magnetic attachments have more advantages compared with bar-clip attachments, such as decreased procedure time, easier cleaning, and lower component costs [13,14,15].

A magnet-retained mini-implant overdenture system was developed; here, we describe a mandibular clinical procedure and evaluate the biomechanical performance.

## 2. Case Presentation

**Figure 1:**
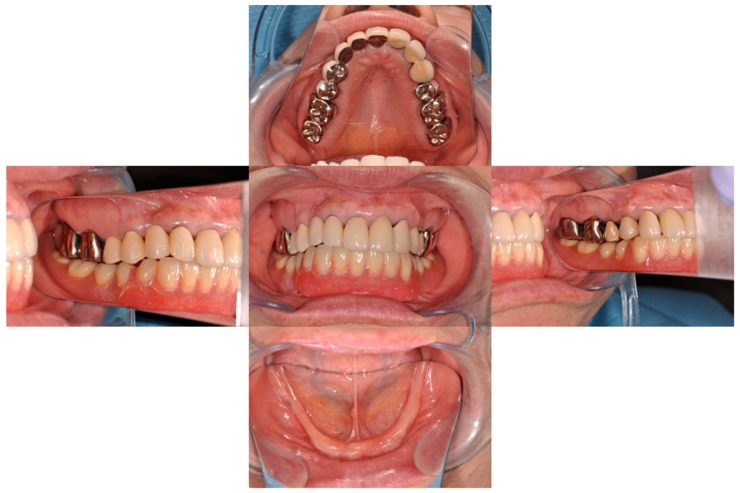
Preoperative view of oral cavity.

A magnet-retained two-mini-implant overdenture was installed in a 67-year-old female patient with natural dentition in the maxilla and total edentulism in the mandible (Figure 1). The patient wore a bilateral fixed partial denture between the canines and a bilateral removable partial denture in the mandible. The lower anterior teeth were extracted due to periodontal disease, and the removable partial denture was affixed to the complete denture. The patient had complete denture instability and needed higher mastication performance and denture stability.

**Figure 2:**
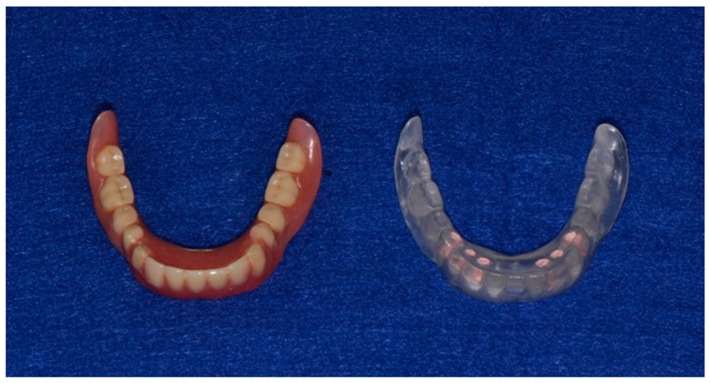
Wearing denture (**Left**) and diagnostic denture embedded guttapercha points in the 432┬234 (34,33,32,42,43,44) regions (**Right**).

**Figure 3:**
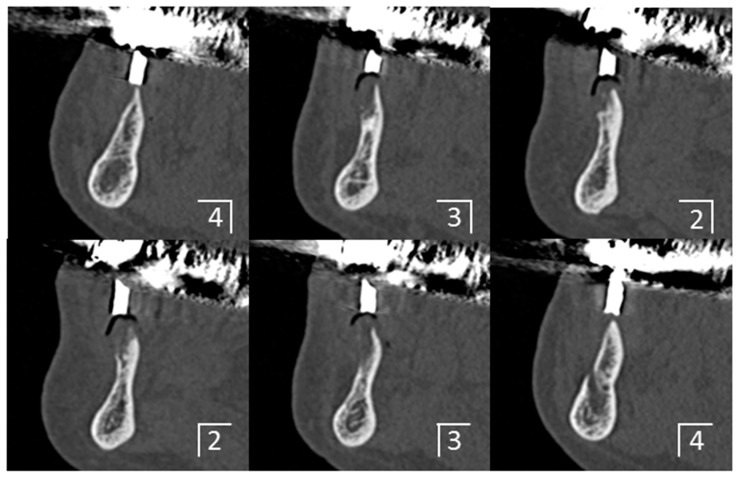
Preoperative computer tomograpy images taken in placing the diagnostic denture. Cross-sectional images at each position.

**Figure 4:**
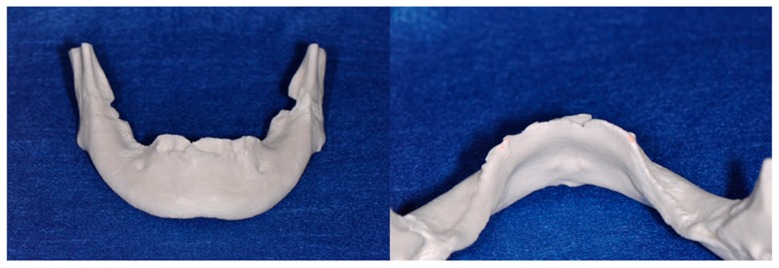
The plaster model of mandible fabricating by a three-dimensional printer on the CT data. Front (**Left**) and rear (**Right**) views.

A medical computer tomography (CT) examination was performed for the placement of a diagnostic denture (Figure 2 and Figure 3), and a plaster model of the mandible was then fabricated from CT data using a three-dimensional printer (Figure 4). The bone shape was examined using the CT images and plaster model, and a thin residual ridge was found around the anterior region.

**Figure 5:**
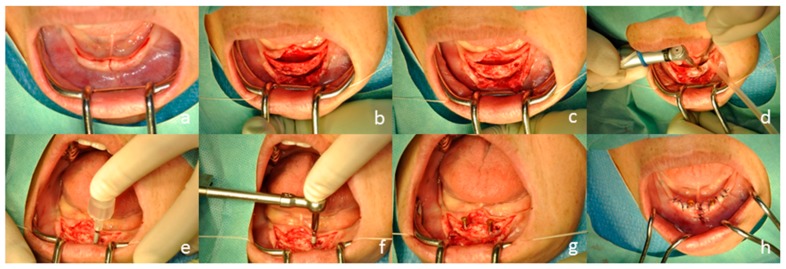
Surgical installation. Incision and periosteal flap was opened. As alveolar plasty is required in the preoperative examination, wider and longitudinal incisions were also added (**a**,**b**); alveolar plasty (**c**) and drilling (**d**) were performed. Two implant cavities were drilled parallel to each other. Importantly, the perforation through lingual cortical bone was avoided. Implant placement proceeded with hand motion (**e**) and torque wrench (**f**); Final torque at the initial fixation was 25 N. Dome-type keepers were placed (**g**); and the flap was closed with suturing (**h**). The implant was positioned so that the top of dome-type keeper was the same height as the mocosa.

**Figure 6:**
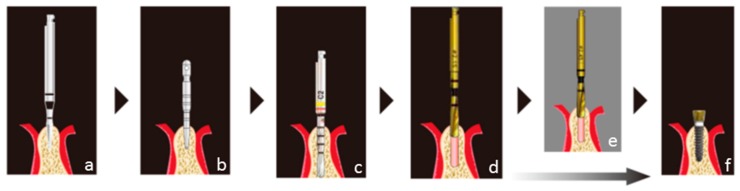
Drilling steps for preparing the implant cavity: Perform cortical bone using a guide drill with less than 700–800 rpm under saline irrigation (**a**); Confirm the direction and position of implant cavity using guide pin (**b**); Prepare the implant cavity using starting drill (φ1.8 mm, **c**) and implant drill (φ2.1 mm, **d**); Use implant drill (φ2.4 mm, **e**); if the bone is dense, and implant placement (**f**). Reproduced with permission from (Aichi Steel, Aichi, Japan) & (Platon Japan, Tokyo, Japan).

Two mini-implants (Magfit^®^ MIP, Aichi Steel, Aichi, Japan & Platon Japan, Tokyo, Japan) were placed in the anterior region of the mandible. After local anesthesia, the periosteal flap was opened and minimal osetoplasty was done prior to placement of the implant. The mini-implants were placed at the planned positions according to the manufacturer’s directions using the preoperative examinations, and a dome-type keeper was connected to the mini-implant (Figure 5 and Figure 6).

**Figure 7:**
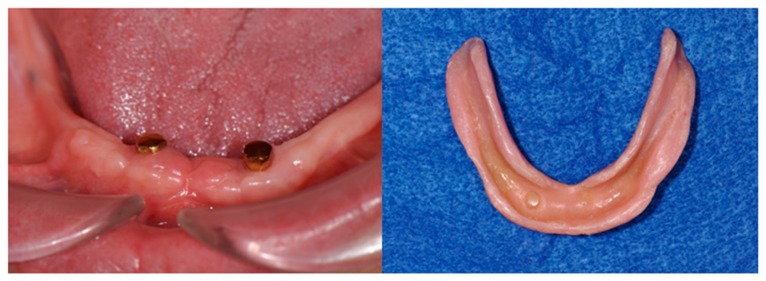
Three-month postoperative view of mandible (**Left**), and the tissue conditioning material was relined under denture base of the wearing denture (**Right**).

After surgical installation, the denture was fixed so that the implant placement region was relieved by the application of a tissue conditioning material (Figure 7).

**Figure 8:**
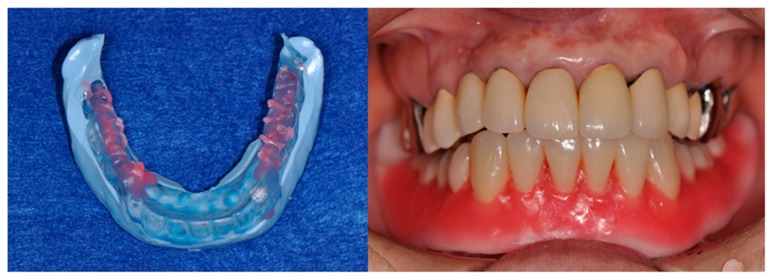
The completion of final impression and interocclusal records using the duplicated denture (**Left**). Wax lower denture was fabricated according to the morphology of duplicated denture (**Right**).

**Figure 9:**
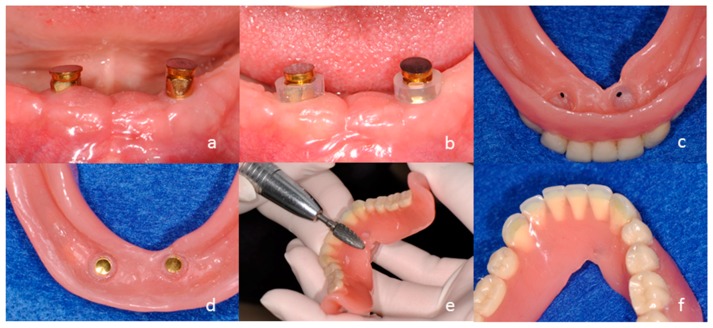
Installation of magnet assembly to the new overdenture using an auto-curing acrylic resin. Place the metal-adhesion-treated magnet assembly (**a**); Set the silicone tube on the keeper (**b**); Make a hole though denture base (**c**); Apply the resin material and fix the magnet assembly to the denture base (**d**); Remove the extra resin (**e**); Polish (**f**). The magnet assemble should be fixed to the denture base with the load, or after the overdenture is settled down to the oral mucosa.

Three months after the procedure, the final impression and interocclusal record of the duplicated denture were obtained (Figure 8). A new magnet-retained two-mini-implant overdenture with hard artificial teeth (ENDURA, Shofu, Kyoto, Japan) was manufactured by conventional denture fabrication. Two magnet assemblies with concave surfaces having a complementary relationship with the dome-type keepers were placed on the mini-implant. The two magnet assemblies were connected to the denture base to hold the complete overdenture in position on the mucosa. Both the mucosa and implants provided support, retention, and stability (Figure 9).

The patient was satisfied with masticatory performance and appearance with the magnet-retained two-mini-implant overdenture.

## 3. Clinical and Mechanical Considerations

A 2-IOD was built as the primary choice for mandibular edentulism [16,17]. In this case, the patient rejected either treatment option of a fixed four-implant bridge due to cost concerns or an additional surgery such as bore grafting before the placement of two regular implants. Therefore, we decided to use only two mini-implants for this case. Although there are a few reports on the clinical outcome of the 2-IOD using mini-implants [18,19], the outcome is still unclear. Mini-implants may cause mechanical overstressing of the occlusion due to the narrow implant body form [20].

The success of implant overdenture depends on three conditions: implant positioning, selection of attachment, and the splinting between the attachment and denture base [21,22]. Mini-implants can be inserted in narrow ridges via minimally invasive surgery, and are appropriately positioned when parallel to each other and perpendicular to the occlusal plane.

We recommend splinting of the implants in the overdenture because of decreasing the stress around the implants [23]. However, even if the implants are splinted, occlusal stress is not evenly distributed between the implants [24]. The center implant is most likely to be affected by stress, and that implant may have a worse prognosis than the adjacent implants [25]. The load control to each implant should be addressed with considerations for occlusal scheme and contact.

The stress of the implant is the inverse square of the implant diameter [26]. Assuming that a regular implant has a diameter of 3.8 mm and a mini-implant has a diameter of 2.7 mm, the stress of a mini-implant will be about twice as large as that of a regular implant. If assumed that the threshold of bone resorption based on Frost’s theory is 1000 με (20 MPa) [27], the occlusal force is supported around the neck of a 3.75-mm-diameter implant with a 2-mm width and bone contact ratio is 50%; thus, the acceptable amount of occlusal force is ~200 N and 100 N for regular- and mini-implants, respectively. In the previous study, maximum bite force in mandibular two-implant overdenture with a bar attachment was reported around 100 N [28]. Considering this fact, we predict that the load against a mini-implant will not exceed 100 N during conventional mastication, but may occur due to denture movement; special care should be paid to this case.

Magnetic attachment is effective for avoiding excessive horizontal force against the implant, but provides no buffering for axial movements and may require gaps between the keeper and the magnet assembly [29]. The magnet assembly should be installed on a denture base using any load greater than 0 N; this is called “after settling down the denture delivery”. Clinically, the role of the mini-implant is to provide slight support and retention, and these functions were maintained during this study.

Implant overdentures require a level of maintenance that far exceeds that of fixed implant denture. We should give constant attention to the occlusal changes although the hard resin artificial teeth with high strength and wear resistance were used for the implant overdenture. In addition, we have to pay attention to the denture movements by making the implants as a rotation fulcrum, as the alveolar ridge under the denture base area is absorbed for a long term. When the implant overdenture loses soft tissue support and begins to move more freely, relining is applied as maintenance, and the magnetic attachment functions successfully for a long time.
